# An Objective Diagnosis Model with Integrated Metabolic and Immunity Parameters for Phlegm-Dampness Constitution

**DOI:** 10.1155/2022/3353549

**Published:** 2022-02-04

**Authors:** Yanchun Huang, Shanshan Guo, Jun Yang, Yangfan Tang, Xinghua Zhu, Sichong Ren

**Affiliations:** ^1^Department of Laboratory Medicine, The First People's Hospital of Longquanyi District, Chengdu, West China Longquan Hospital Sichuan University, Chengdu 610100, China; ^2^Center for Translational Medicine, Sichuan Academy of Traditional Chinese Medicine, Translational Chinese Medicine Key Laboratory of Sichuan Province, Chengdu 610041, China; ^3^Department of Nephrology, Clinical Medical College and the First Affiliated Hospital of Chengdu Medical College, Chengdu, China

## Abstract

**Background:**

According to Chinese constitutional theory, people can be divided into nine constitutions, which represent distinctive vulnerability to different diseases such as metabolic syndrome, atherosclerosis, and immunity-related disease, and so forth in modern medicine, phlegm-dampness constitution (PDC) is one of the nine constitutions, which is susceptible to metabolic syndrome (MS) and atherosclerosis that associate with lipid metabolism and immunity dysregulation closely.

**Objectives:**

In this study, we aimed to investigate the metabolic and immunity profiles of phlegm-damp constitution (PDC), including metabolites, lymphocytes distribution, and inflammatory cytokines.

**Methods:**

A total of 74 patients with PDC and 66 individuals with gentle constitution (GC) were enrolled in this study. We utilized biochemical methods to detect metabolic parameters, flow cytometry to survey T/B/NK/NKT lymphocyte subgroups distribution, and ELISA to assay inflammatory cytokines.

**Results:**

The subjects with PDC had higher GLU, AI TC, TG, and LDL-C and lower HDL-C levels. The immunity profile indicated that PDC subjects had higher percentage of WBCs, neutrophils, lymphocytes, B cells, and natural killer T cells compared with subjects with GC (*P* < 0.05). Serum levels of IL-10 decreased significantly in the subjects with phlegm-damp constitution, whereas IL-12 levels increased dramatically in the PDC group compared with the GC group (both *P* < 0.05). Additionally, logistic regression identified four independent variables (GLU, TG, LDL-C, and lymphocytes) that were highly correlated with PDC (*P* < 0.05). The area under the curve of the receiver operating characteristic curve was 0.878, which indicated the data were reliable to distinguish the subjects with PDC from the ones with GC.

**Conclusion:**

Phlegm-damp constitution was prone to hyperglycemia and hyperlipidemia syndrome, promoting the occurrence and progression of metabolic-related diseases. Interestingly, proinflammatory cells and cytokines were activated in the PDC group as well. Our findings could offer a profile of early screening indicators to identify high-risk patients of metabolic- and immunity-related diseases from Chinese constitution.

## 1. Introduction

Chinese constitutional theory was proposed in the 1970s and has been widely used in clinical practice by traditional Chinese medical (TCM) doctors for disease management and health promotion [1]. According to this theory, people are divided into nine constitutions, which comprise one balanced constitution (normality) and eight imbalanced constitutions (phlegm-dampness, damp-heat, yang-deficient, blood stasis, yin-deficient, qi-deficient, qi-stagnation, and special intrinsic constitution) based on the Classification and Determination of Constitution [2]. Constitution is an individual life-related stable phenotype, which is considered as an integrated indicator of physical and psychological characteristics individually affected by hereditary and acquired environment together. A plenty of studies from TCM has showed that it tendentiously determined an individual's susceptibility to certain diseases, and it was regarded as a risk factor of its related diseases. Eventually, these imbalanced constitutions could initiate and accelerate different diseases [3].

Phlegm-dampness constitution (PDC) is one of the eight imbalanced constitutions and most common type in TCM studies. The following are the common characteristics of PDC: fat, greasy coating on the tongue, slippery pulse, copious and sticky sweat, dark yellow facial complexion, face and eyelid edema, copious phlegm, drowsiness, heavy limbs, etc. [3]. People with PDC are unable to adapt to the moist environment and vulnerable to dampness syndrome. Studies demonstrated that PDC patients are susceptible to diabetes, metabolic syndrome (MS), and cardiovascular disease (CVD) [4, 5]. MS is termed as a group of interrelated cardiometabolic risk factors, including elevated fasting glucose, elevated triglycerides (TGs), reduced high-density lipoprotein (HDL), elevated blood pressure, and central obesity [6]. Approximately 30% of adults had MS worldwide, and the prevalence of the disease was increased in various countries and regions recently [7, 8]. Studies demonstrated that MS contribute to CVD in patients remarkably [9, 10]. In TCM theory, PDC is considered as the early stage of MS. Therefore, diagnosis and intervention of PDC are important to decrease the incidence of MS and CVD.

Immunity plays a critical role in the occurrence and development of MS, diabetes, and CVD [11–13]. TCM suggested that dysfunction of body's spleen and stomach could affect the metabolism and immunity balance, which gradually become PDC [14]. Similarly, modern medicine confirmed that the spleen is a vital organ of immune cell development and immune function regulation [15]. Hitherto, there is no report about immunological profile including immune cell subtypes and cytokine parameters in PDC subjects.

Thus, this study investigated lipid metabolites, lymphocyte subtypes distribution, and inflammatory cytokines in PDC. Results had indicated that lipid metabolism was dysregulated and accompanied with a higher percentage of B cells and natural killer T cells in PDC patients. It offered us a point of connecting PDC with CVD in these two medicine theories, meanwhile, which provided novel ideas for objective screen and intervention of PDC in future.

## 2. Methods

### 2.1. Patients

All subjects aged 35–84 years were selected from the outpatients of the Cardiology Department and Health Management Center, West China Longquan Hospital Sichuan University, from January 2019 to June 2020. The subjects were judged independently by two experienced doctors who calculated scores according to the criteria evaluation of Chinese constitution [3]; for example, white-greasy tongue coating (5 scores) and swollen tongue proper (4 scores) with slippery pulse (4 scores). The phlegm-dampness-related symptoms include the sensation of tiredness, listlessness, and sick feeling (3 scores), slightly edematous eyelids (2 scores), chest stuffiness (3 scores), sleepiness (2 scores), fatty and soft abdomen (2 scores), abundant mucous or thin white sputum (1 score), swollen lower limbs (2 scores), pale-yellow complexion (1 score), and slimy sensation in the mouth (1 score). Individuals with total scores >9 were judged as PDC. However, gentle constitution (GC) showed some symptoms, such as calm, strong pulse, and pink tongue with thin white coating, with total score <9 according to Chinese constitution criteria. In this study, the inclusion criteria of PDC are outlined as follows: (a) transformed scores of the phlegm-dampness-related symptoms are greater than 40; and (b) two doctors have no controversy. The inclusion criteria of GC are outlined as follows: (a) transformed scores of are greater than 60 and transformed scores of imbalanced constitutions are less than 30; and (b) two doctors have no controversy. Subjects were excluded if they exhibited any of the following: (a) two doctors have a controversy; (b) subjects with other autoimmune diseases, cancer, systemic diseases, and other serious diseases; or (c) subjects in the acute phase of the disease (for example, infection).

### 2.2. Main Reagents and Equipment

The main reagents and equipment used in this study were as follows: automatic blood cell counter BC6900 and supporting reagents (Mindray, China); automatic biochemical apparatus type AU5800 (Beckman Coulter, USA), and biochemical reagent (Orienter, China); flow cytometry DxFLEX6 and antibodies for lymphocytes identification (Beckman Coulter Life Sciences); ELISA for cytokine testing Kits (R&D).

### 2.3. Leukocyte Count

Blood from subjects was collected in a tube with EDTA (anticoagulant) and assessed using the automatic blood cell counter BC6900, according to the standard operating procedure (SOP).

### 2.4. Biochemical Measurement

Whole blood from subjects was collected, and their serum was isolated. Fasting blood glucose (GLU), total cholesterol (TC), triglyceride (TG), low-density lipoprotein-cholesterol (LDL-C), high-density lipoprotein-cholesterol (HDL-C), apoprotein A (apoA), and apoprotein B (apoB) were measured by the colorimetric method on an automatic biochemical apparatus type AU5800 (Beckman Coulter), according to its SOP. The arteriosclerosis index (AI) was calculated using the formula (TC − HDL)/HDL.

### 2.5. Flow Cytometry Analysis

Peripheral blood samples were obtained from subjects. For antibody staining, 10 mL of the indicated antibody was mixed gently to 100 mL blood sample and incubated at room temperature in the dark for 15 min. Subsequently, 500 mL hemolysin buffer was added to the same sample tube and mixed gently and incubated at room temperature in the dark for an additional 15 min, followed by an addition of 500 mL sheath buffer for detection. The indicator antibodies were as follows: CD45-FITC/CD4-RD1/CD8-ECD/CD3-PC5 was used for T/B lymphocyte subsets (6607013, Beckman Coulter Life Sciences), and CD45-FITC/CD3-RD1/CD19-ECD/CD56-PC5 was used for B/natural killer (NK)/NKT lymphocyte subset (6607073, Beckman Coulter Life Sciences). The distribution of all the circulating T, B, NK, and NKT cell subsets was assessed by flow cytometry (DxFLEX6; Beckman Coulter Life Sciences) according to the manufacturer's protocol. The threshold for positive staining was determined using fluorescence minus one (FMO) control. Additionally, the performance of the instrument was verified daily using the tracking system to ensure the consistency of flow cytometry.

### 2.6. Cytokines Measurement

Blood samples were collected from subjects, and levels of IL-4, IL-2, IFN-g, IL-6, IL-10, and IL-12 were detected using commercial ELISA kits (R&D), according to the manufacturer's instructions.

### 2.7. Statistical Analysis

Statistical analysis was performed using SPSS Statistics 22.0. for Windows (SPSS, Inc., Chicago, IL, USA). All figures were constructed using Prism software (GraphPad Prism 7). The values were expressed as mean ± SD or median (25^th^–75^th^ percentile). For parametric data, we used the independent samples *t*-test, while nonparametric data were evaluated using the Mann–Whitney *U* test. Pearson's correlation coefficient was used to assess correlations. Univariate logistic regression analysis was performed for all indexes associated with PDC. The identified variables for which the *P*-value <0.05 in the univariate analysis were further assessed with binary logistic regression (stepwise forward), and the probability of having PDC was obtained. Then, probability estimates were used to plot the receiver operating characteristic (ROC) curve. We used the Hosmer–Lemeshow goodness-of-fit test to assess model fit. The equation of this model is *P* (*y* = 1) = *e*^*y*^/(1 + *e*^*y*^), *y* = *β*0 + *β*1*X*1 + *β*1*X*1 + *β*2*X*2 + *β*3*X*3 + … + *βnXn*, *e* = 2.718. For all the tests, the statistical significance was set at *P* < 0.05.

## 3. Results

### 3.1. Metabolites and Routine Blood Testing Parameters of Recruited Subjects

Among recruited subjects, 74 patients had PDC and 66 volunteers were controls (gentle constitution). The cohort comprised 56.8% and 51.5% females in PDC and GC groups, respectively. As shown in Table 1, the group of PDC had higher diabetes prevalence and cardiovascular disease prevalence than the group of gentle constitution. The median age was 51.20 ± 11.80 years and 49.41 ± 11.55 years in PDC and GC groups, respectively. The two groups were similar in terms of age and gender. The PDC group had higher levels of GLU, AI, TC, TG, LDL-C, WBC, lymphocyte counts, and neutrophil counts from routine blood testing but a lower HDL-C level in lipid metabolism (Table 1).

### 3.2. Lymphocytes Subpopulations Distribution

**Figure 1:**
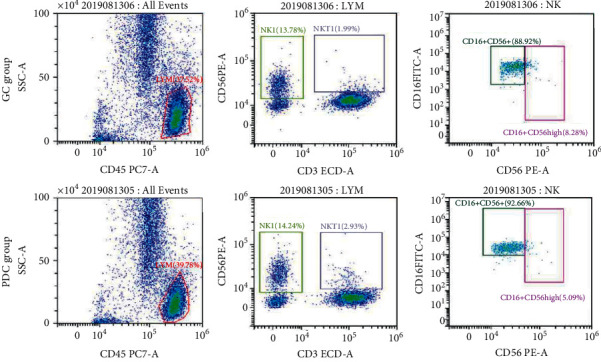
Lymphocyte subpopulations distribution in PDC and GC groups. The left lane two photos represent total white cells, and these marked cell clusters by red circles indicate lymphocytes. The medial lane two photos represent NK and NKT cells. The right lane two photos represent CD16^+^CD56^+^ and CD16^+^CD56^+high^.

Flow cytometry was applied to investigate the distribution of leukocyte subpopulations in the two groups. Compared with the GC group, the PDC group had higher events of lymphocytes, B cells, and NKT cells (*P* < 0.05). Percentages of B and NKT cells were higher in the PDC group than GC group. However, the GC group had a higher percentage of total lymphocytes than the PDC group. Besides, percentages of T and NK cells were similar in both groups (Table 2). These cytometry photographs of lymphocytes distribution displayed lymphocyte subsets, NKT and NK cells, and effector NK (CD16^+^CD56^+high^) representing immunity states of the two groups (Figure 1).

### 3.3. Serum Cytokine Levels

**Figure 2:**
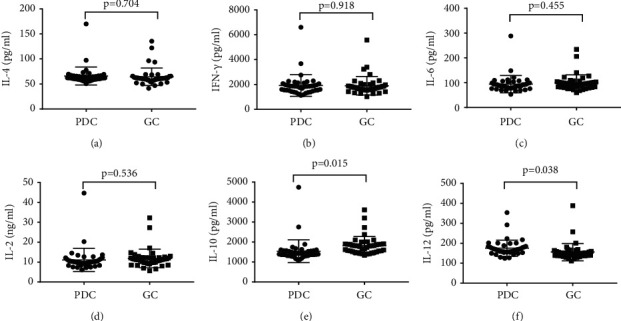
Inflammatory cytokines in the serum of PDC and GC groups. The scatter plot of cytokines for PDC (phlegm-damp constitution) and GC (gentle constitution). IFN-*γ*: interferon *γ*. *P* values less than 0.05 represent significant difference.

Serum IL-4, IL-2, IL-6, IL10, IL-12, and IFN-*γ* cytokine levels reflect inflammatory states; we investigated these inflammatory cytokines in PDC and GC groups (Figure 2). In this study, the serum levels of anti-inflammatory cytokine IL-10 decreased significantly in the PDC group compared with the GC group (*P*=0.015). However, the proinflammatory cytokine IL-12 level increased dramatically in the PDC group compared with GC group (*P*=0.038). Other cytokines IFN-*γ*, IL-4, IL-2, and IL-6 showed no difference between the PDC and GC groups.

### 3.4. Logistic Regression Model for PDC

**Figure 3:**
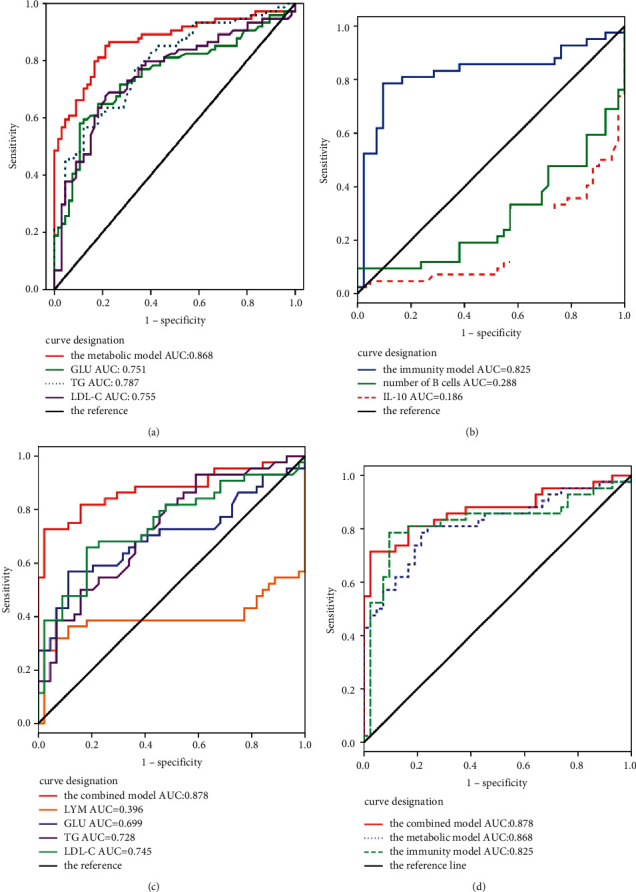
Area under the receiver operating characteristic curve. (a) ROC analysis for the metabolic model and the variables of GLU (fasting blood glucose), TG (triglyceride), and LDL-C (low-density lipoprotein-cholesterol). AUC = area under the ROC curve. (b) ROC analysis for the immunity model and the variables of number of B cells and IL-10. (c) ROC analysis for the combined model and the variables of GLU, TG, LDL-C, and LYM (lymphocyte). (d) ROC analysis for the combined model, the metabolic model, and the immunity model.

Logistic regression analyses (stepwise, forward) were used to establish three identifiable PDC models. There were four independent variables in the combined model: GLU (odds ratio (OR): 1.470, 95% CI: 1.069–2.021), TG (OR: 1.759, 95% CI: 0.897–3.450), LYM (OR: 0.858, 95% CI: 0.773–0.952), and LDL-C (OR: 2.512, 95% CI: 1.193–5.289) (Table 3). GLU (OR: 1.470, 95%CI: 1.154–1.873), TG (OR: 2.138, 95%CI: 1.370–3.337), and LDL-C (OR: 2.295, 95%CI: 1.300–4.054) were included as covariates in the metabolic model (Table 3). In the immunity model, the number of B cells (OR: 0.993, 95% CI: 0.988–0.999) and IL-10 (OR: 0.999, 95%CI: 0.998–1.000) were include (Table 3). The Hosmer–Lemeshow goodness-of-fit test was also implemented, which indicated that there was no overfitting of the models. Besides, we drew the receiver operating characteristic curve to check sensitivity and specificity parameters. The results showed that the area under the curve of the metabolic model, the immunity model, and the combined model were 0.868, 0.825, and 0.878, respectively (Figure 3). The sensitivity and specificity of the combined model were 0.727 and 0.977, respectively, when the cutoff was 0.610 (Table 4).

## 4. Discussion

In this study, we found that the PDC group had higher glucose, TG, TC, and LDL-C levels compared with the gentle constitution group. Some investigators found that imbalanced constitutions, especially PDC, had a close correlation with abnormal glucose and dyslipidemia [16–18]. In addition, researchers have explained these phenotypes from the molecular level that five genes and six SNPs were identified differently between PDC and GC. These genes and SNPs were involved in gluconeogenesis and thermoregulation, as well as the process of lipid metabolism and brown fat cells differentiation, which indicated that individuals with PDC were susceptible to metabolic disorders from the genetic level [19, 20]. We found that B cells were significantly higher in the subjects with PDC compared with that of GC. Some scholars suggested that B cells played a central role in the development of insulin resistance, which could make an interpretation of obesity-associated insulin resistance caused by activation of B cells [21]. Many evidence also supported the activation of B cells was significantly higher in obese patients [22]. Increased B cells in PDC may be accounted for the obese characteristic of PDC dysregulated B cell immunity, which contributed to diabetes mellitus. The existing literature suggests that increased lymphocyte counts are associated with decreased insulin sensitivity and higher risk to develop diabetes [23]. Furthermore, individuals with DM have increased memory CD4+ T cells populations and decreased regulatory T cells, which create a proinflammatory state [24, 25]. In conclusion, these data suggest that patients with diabetes may have higher lymphocytes characterized by a functional profile skewed towards a proinflammatory state. In our study, the individuals with PDC had higher lymphocyte counts than the ones with GC, suggesting our results of the present study were consistent with these previous studies.

NKT cells are a nonconventional subtype of CD4^+^ T cells, which firstly described in mice by Bendelac in 1994 [26]. Its cellular surface markers are constituted by NK cell marker CD56^+^ and T cell receptors together. Usually, it recognizes lipid molecules via CD1d but not by major histocompatibility complex (MHC) presenting. Therefore, NKT cells are good executors, which orchestrate innate immune, adaptive immune, and lipid metabolism finely [27]. It has been shown that circulating NKT cells in obese patients were lower than healthy controls [28]. Researchers presumed that a number of NKT cells were stored in adipose tissue [29], so that it could recognize endogenous glycolipids and phospholipids easily to play a critical role in metabolic inflammation modulation [30–32]. Recently, a few studies have suggested that NKT cells played an important role in human atherosclerosis development [33, 34]. Here, we found NKT cell expansion increased in PDC, which may be contributed to diabetes and metabolic syndromes at early stage for initiating metabolic inflammatory cascades in TCM theory. Undeniably, NKT cells play a major role in immunoregulation of PDC, which accounts for that the ones with PDC are especially susceptible to ending with MS and CVD. More detail mechanisms of NKT regulating PDC outcomes need to be elucidated in future.

Chronic inflammation is a crucial culprit for development of diabetes mellitus and cardiovascular diseases [35, 36]. Inflammation is finely regulated by proinflammatory cytokines and anti-inflammatory cytokines coordinately. In this study, we found that the anti-inflammatory cytokine IL-10 was decreased, whereas proinflammatory IL-12 was increased in the PDC group. Studies demonstrated that IL-10 could improve impaired insulin signaling caused by proinflammatory cytokines [37]. In vivo studies showed that IL-10 could prevent IL-6 or lipid-induced insulin resistance [38]. Moreover, IL-10 was testified to be decreased in MS as well [39]. These previous data support the finding that IL-10 could be a negative biomarker of PDC. Inversely, the proinflammatory IL-12 may be an indicator of PDC and its complications.

Hitherto, PDC is diagnosed mainly according to doctors' consulting experience. So, objective markers and quantitative parameters are needed urgently to offer a more reliable diagnosis of it. Studies have indicated that metabolic disorders including glucose, lipid, and cholesterol were tightly associated with PDC, which could be good indicators of it [40, 41]. In our study, we found lymphocyte counts, the number of B cells, and the number of NKT cells changed dramatically between PDC and GC. Therefore, we have tried to establish a diagnostic model based on biochemical parameters and immune cell subsets, which might be more effective for diagnosing and managing of people with PDC. In this combined model, the variables GLU, TG, LDL-C, and lymphocyte counts were finally included, and the area under the curve was 0.878. Interestingly, another group considered PDC as a risk factor of MS and established a diagnostic model including nine related parameters, such as blood sugar, triglycerides, and blood pressure [42]. The area under the curve was 0.865 in their model, which was consistent with our results. In this study, we firstly integrated metabolic and immunity parameters to set an evaluation model distinguishing the ones with PDC from the individuals confused as GC, or other constitution, which might be more suitable for the early-stage screening and health management of individuals with PDC.

In this study, more volunteers should be recruited to improve the representative and accuracy of the diagnostic model, while small sample size might reduce the power of model testing. To remedy this shortcoming, our data were collected by trained staff using standardized instruments according to standardized operating procedures (SOP).

## 5. Conclusion

Phlegm-damp constitution is especially vulnerable to diabetes, MS, and CVD compared with GC. Metabolic inflammation is a key culprit for triggering these metabolic-related diseases. Here, we firstly integrated metabolic and immunity parameters to establish an objective model for PDC diagnosis and management. It could help screen high-risk patients in the early stages of PDC and offer an evidence-based method for TCM clinical and basis research.

## Figures and Tables

**Table 1:** Metabolites routine blood testing parameters of subjects

Parameters	PDC (*n* = 74)	GC (*n* = 66)	*P*
Sex(male/female)	32/42	32/34	0.534
Age, years	51.20 ± 11.80	49.41 ± 11.55	0.265
Diabetes (%)	31 (41.89%)	7 (10.61%)	**<0.001**
Receiving glyburide monotherapy (%)	4 (5.41%)	1 (1.54%)	0.221
History of coronary artery disease	10 (15.63%)	1 (1.54%)	**0.008**
Receiving atorvastatin monotherapy (%)	1(1.35%)	0(0.00%)	0.529
GLU, mmol/L	7.73(5.01–12.81)	5.26 ± 1.29	**<0.001**
AI	5.17 (2.56–4.48)	2.33 (1.90–2.83)	**0.024**
TC, mmol/L	5.87 (4.81–6.52)	4.63 ± 0.96	**0.001**
TG, mmol/L	3.81 (1.84–3.81)	1.65 ± 0.80	**<0.001**
HDL-C, mmol/L	1.27 ± 0.43	1.44 ± 0.36	**0.012**
LDL-C, mmol/L	3.75 (2.93–4.17)	2.77 ± 0.64	**0.001**
apoA, g/L	1.16 ± 0.27	1.25 ± 0.25	0.093
apoB, g/L	1.12 ± 0.34	1.07 ± 1.01	0.753
WBC, ×10^9^/L	7.29 (5.54–7.54)	6.24 (4.86–7.54)	**0.002**
N, ×10^9^/L	4.80 (3.37–5.44)	3.55 (2.78–4.99)	**<0.001**
LYM, ×10^9^/L	1.98(1.47–2.51)	1.78 ± 0.76	**0.028**
NLR	2.37(1.88–3.08)	1.94 (1.25–2.24)	0.302
PLT, ×10^9^/L	182.20 ± 56.30	184.26 (166.00–216.50)	0.832
PLR	125.59 (65.50–140.50)	98.7(74.00–135.00)	0.298
RBC, ×1012/L	6.41 ± 16.33	4.60 ± 0.65	0.374
HCT	42.03 (38.2–47.35)	41.91 ± 5.19	0.912
HB, g/L	137.23 ± 18.99	140.12 ± 15.77	0.339

PDC: phlegm-damp constitution, GC: gentle constitution, GLU: fasting blood glucose, AI: arteriosclerosis index, TC: total cholesterol, TG: triglyceride, HDL-C: high-density lipoprotein-cholesterol, LDL-C: low-density lipoprotein-cholesterol, apoA: apoprotein A, apoB: apoprotein B; N: neutrophils; LYM: lymphocytes, NLR: the ratio of neutrophil/lymphocyte, PLT: platelets, PLR: the ratio of platelet/lymphocyte, RBC: red blood cell, HCT: hematocrit, HB: hemoglobin. Bold font indicates *P* value less than 0.05.

**Table 2:** The levels of mononuclear cells in blood.

Variables	PDC (*n* = 74)	GC (*n* = 66)	*P*
Lymphocytes, %	27.81 ± 3.74	24.23 ± 9.39	**0.021**
Lymphocytes, events/*µ*l	2155.32 ± 619.99	2096.88 ± 907.99	**0.425**
B, %	9.17 ± 2.91	7.03 ± 3.99	**0.005**
B, events/*µ*l	198.86 ± 73.56	142.15 ± 98.10	**0.003**
T, %	71.40 ± 10.46	68.50 ± 7.22	0.134
T, events/*µ*l	1492.80 ± 686.28	1499.09 ± 375.83	0.958
NKT, %	6.46 ± 2.44	4.95 ± 3.86	**0.031**
NKT, events/*µ*l	114.93 ± 105.00	112.41 ± 75.55	**0.023**
NK, %	17.56 ± 9.37	19.09 ± 5.90	0.363
NK, events/*µ*l	371.35 ± 245.71	419.74 ± 173.68	0.289

PDC: phlegm-damp constitution, GC: gentle constitution, B: B lymphocytes, T: T lymphocytes, NKT: natural killer T (NKT) cells, NK: natural killer cells. % means percentage of cells. Events/*µ*l indicates counting of cell absolutely. *P* values less than 0.05 represent significant difference.

**Table 3:** Variables used in different models.

Model	Variables	*β*	SE	Wald	*P*	OR (95% CI)
The metabolic model	GLU	0.385	0.123	9.754	0.002	1.470 (1.154, 1.873)
	TG	0.76	0.227	11.193	0.001	2.138 (1.370, 3.337)
	LDL-C	0.831	0.290	8.194	0.004	2.295 (1.300, 4.054)
	Constant	−6.619	1.253	27.905	<0.001	0.001
The immunity model	Number of B cells	−0.007	0.003	5.208	0.022	0.993 (0.988, 0.999)
	IL-10	−0.001	0.001	2.684	0.101	0.999 (0.998, 1.000)
	Constant	2.987	1.142	6.844	0.009	19.828
The combined model	LYM	−0.153	0.053	8.391	0.004	0.858 (0.773, 0.952)
	GLU	0.385	0.162	5.63	0.018	1.470 (1.069, 2.021)
	TG	0.565	0.344	2.697	0.101	1.759 (0.897, 3.450)
	LDL-C	0.921	0.380	5.884	0.015	2.512 (1.193, 5.289)
	Constant	−2.681	1.846	2.109	0.146	0.068

*β*: regression coefficient, SE: standard error, Wald: Wald chi-square value, CI: confidence interval, OR: odds ratio, GLU: fasting blood glucose, TG: triglyceride, LDL-C: low-density lipoprotein-cholesterol, B: B lymphocytes, LYM: lymphocytes. *P* values less than 0.05 represent significant difference.

**Table 4:** Performance of the models distinguishing subjects with PDC from subjects with GC.

Models^1^	AUC (95% CI)	*P* ^2^	Sensitivity	Specificity	Cutoff^3^
The metabolic model	0.868 (0.806, 0.929)	<0.001	0.851	0.788	0.433
The immunity model	0.825 (0.727, 0.924)	<0.001	0.786	0.905	0.563
The combined model	0.878 (0.801, 0.954)	<0.001	0.727	0.977	0.610

^1^The models were constructed by logistic regression analyses (stepwise, forward). ^2^*P* values for the significance of differences between AUCs and 0.5 by the Wilcoxon rank-sum test. ^3^The optimal cut-point values in ROC (the receiver operating characteristic curve) analyses were defined by the Youden index. PDC: phlegm-damp constitution, GC: gentle constitution, CI: confidence interval, AUC: the area under the curve.

## Data Availability

Data of the figures and tables used to support the findings of this study are included within the article.
